# QTL Detection and Candidate Gene Identification for Eating and Cooking Quality Traits in Rice (*Oryza sativa* L.) via a Genome-Wide Association Study

**DOI:** 10.3390/ijms25010630

**Published:** 2024-01-03

**Authors:** Jianhua Jiang, Shaojie Song, Changmin Hu, Chunyu Jing, Qing Xu, Xinru Li, Mengyuan Zhang, Mei Hai, Jiaming Shen, Ying Zhang, Dezheng Wang, Xiaojing Dang

**Affiliations:** 1 Anhui Province Key Laboratory of Rice Genetics and Breeding (Rice Research Institute), Anhui Academy of Agricultural Sciences, Hefei 230031, China; 2 College of Agronomy, Anhui Agricultural University, Hefei 230036, China

**Keywords:** candidate genes, eating and cooking quality, gene expression, genome-wide association study, quantitative trait loci, single nucleotide polymorphism

## Abstract

The eating and cooking quality (ECQ) directly affects the taste of rice, being closely related to factors such as gelatinization temperature (GT), gel consistency (GC) and amylose content (AC). Mining the quantitative trait loci (QTLs), and gene loci controlling ECQ-related traits is vital. A genome-wide association study on ECQ-related traits was conducted, combining 1.2 million single nucleotide polymorphisms (SNPs) with the phenotypic data of 173 rice accessions. Two QTLs for GT, one for GC and five for AC were identified, of which two were found in previously reported genes, and six were newly found. There were 28 positional candidate genes in the region of *qAC11*. Based on a linkage disequilibrium (LD) analysis, three candidate genes were screened within the LD region associated with AC. There were significant differences between the haplotypes of *LOC_Os11g10170*, but no significant differences were found for the other two genes. The qRT-PCR results showed that the gene expression levels in the accessions with high ACs were significantly larger than those in the accessions with low ACs at 35d and 42d after flowering. Hap 2 and Hap 3 of *LOC_Os11g10170* reduced the AC by 13.09% and 10.77%, respectively. These results provide a theoretical and material basis for improving the ECQ of rice.

## 1. Introduction

Rice is one of the most important crops in the world, feeding nearly one-third of the world’s population. With the increase in rice yield and the improvement of people’s living standards, the pursuit of rice quality has been paid more and more attention to. Rice quality mainly refers to the appearance quality and the eating and cooking quality (ECQ), with gelatinization temperature (GT), gel consistency (GC), and amylose content (AC) being the main indicators for measuring the ECQ of rice. Because it is difficult to determine GT directly, the alkali elimination method is usually used to evaluate it indirectly [1]. GT is related to the amount of water and time needed to cook rice. High-GT rice requires more water and a longer cooking time than low-GT rice. High-GT rice is usually used to make canned rice flour or storable cakes, while medium-low-GT rice is generally used for cooking. Xu et al. [2] first analyzed the heredity of GT by using the quality–quantity genetic model and analysis method for endosperm traits. The results showed that GT was controlled by one major gene and many minor genes. *Starch synthase IIa* (*SSIIa*) is a major gene controlling the GT in rice [3]. The *alkali digestion* (*ALK*) gene was isolated via map-based cloning. It is mainly responsible for extending short-branched chains of amylopectin (A + B1 chain) and synthesizing medium-length-branched chains (B2 + B3 chain) [4,5]. Umemoto et al. [6] performed haplotype analysis and found that GT was mainly controlled by three SNP combination types in exon 8 and that the allelic variation types were mainly divided into A-GC, G-GC, and G-TT combination types. The allelic variation in the *ALK* gene caused the difference in the amylopectin structure, which led to the difference in the GT in rice [4,7]. Bao et al. [8] and Waters et al. [9] detected that variations in GT/TT in exon 8 greatly influenced the variation in GT. Fujita et al. [10] indicated that *SSIIa* not only acts on the elongation of long amylopectin chains but also regulates the expressions of *SSI*, *granule-bound starch synthase* (*GBSS*), and *ADP-glucose pyrophosphorylase* (*AGP*). Yang et al. [11] suggested that the interactions of *waxy* (*Wx*) and *SSIIIa* genes had a significant effect on the AC and GT in rice.

Starch accounts for more than 90% of the dry weight of endosperm in milled rice, and its composition and structure are the most important factors determining the ECQ of rice [12]. *GBSSI*, encoded by a *Wx* gene, is mainly responsible for the production of amylopectin, and its allelic variation determines the amylopectin content in rice [13,14,15,16]. In waxy rice, the 23-bp deletion in exon 2 results in the premature termination of *Wx* transcription [17]. Among non-waxy varieties, *Wx* mainly differentiates into the *Wx*^a^ and *Wx*^b^ allelic types, with *Wx*^a^-carrying rice having a high amylose content (more than 25%). In contrast with *Wx*^a^, the variation in *Wx*^b^ is caused by a G-T variant at the splicing site of the intron 1. This variant reduces the splicing efficiency of the premise mRNA, consequently reducing the amount of *GBSSI*, resulting in a lower amylose content [18]. Mikami et al. [19] cloned the *Wx* allele, demonstrating that the A–C variation occurring at exon 6 reduces the amylose content to intermediate levels (18–22%). Chen et al. [20] considered that *Wx* gene variation mainly affected GC and AC, with some impact on GT. *ALK* gene variation mainly affected GT, but had no significant effect on AC.

*Starch branching enzyme* (*SBE*) is the only enzyme in starch synthetase that catalyzes dextran chain branching. Yan et al. [21] found that the *SBE1* and *SBE3* genes had different effects on rice quality under different *Wx* allelic backgrounds. The differences in the genetic effects of the *SBE1* and *SBE3* alleles from different sources may be masked by *Wx* genes. Zhu et al. [22] obtained transgenic rice materials with an AC close to 50% and resistant starch content of approximately 13% by simultaneously down-regulating the expressions of *SBE1* and *SBEIIb*, which were used for diabetic food. These results indicated that different allelic combinations of *SBE* genes could improve ECQ and meet the need for breeding specialized functional rice.

*Debranching enzyme* (*DBE*) genes have a strong effect on the formation of rice quality. Mutations or changes in their expression can affect starch accumulation in the seed endosperm, resulting in a sugar endosperm phenotype [23]. Seven rice endosperm mutant genes have been reported: *Flo1* [24], *Flo2* [25], *Flo3* [26], *Flo4* [27], *Flo5* [28,29], *Flo6* [30], and *Flo7* [31,32]. Among them, *Flo3* produces a floury endosperm and reduces the 16 KD globulin content [26]. *Flo5* causes the missing of the starch synthesis gene *OsSSIIIa* [28,29]. *Flo6* encodes a protein that combines with starch isomerase to regulate starch synthesis and cooperates with *isoamylase 1* (*ISA1*) in the amylopectin synthesis [30]. *ISA3* is mainly involved in the synthesis and degradation of transient starch and has little relationship with stored starch [33]. Zhu et al. [34] found that the transcription factors (*Oryza sativa ethylene-responsive element binding protein 89*) *OsEBP89* and *OsEBP5* can activate *Wx* gene expression in a complex form. Liu et al. [35] demonstrated that the GBSSI protein encoded by *Wx* can function as an oligomer. Zhang et al. [36] indicated that some QTLs can stabilize the splicing efficiency of *Wx* under high-temperature conditions, thus enabling high-temperature insensitive AC expression in rice. *RSR1* (*rice starch regulator 1*) is a key transcription factor involved in the negative regulation of starch-synthesis-related gene (*SSRG*) expression [37]. *OsbZIP58* is capable of directly binding to the *Wx*, *SSSIIa*, *SBE1*, *SBE1b,* and *ISA2* promoters, regulating the expression of these genes. Mutations in *OsbZIP58* result in an increase in short amylopectin chains, a decrease in medium-long chains, and the manifestation of chalkiness [38]. OsbZIP58 interacts with another transcription factor, *lesions stimulating disease resistance 1* (*OsLSD1*), and may affect grain starch accumulation by regulating gibberellin synthesis [39].

Although the functions of some *SSRGs* have been well studied, ECQ is a comprehensive trait and a complex network controlled by many *SSRGs*. Via different allele haplotype analyses and transgenic validation, Tian et al. [13] found that *Wx* and *SSSII-3* could interact to affect AC, GC, and GT, where *Wx* was dominant over AC and GC, while playing a minor role in influencing GT. Therefore, finding more favorable allelic variations in *SSRGs* is of great practical significance in improving rice quality. In this study, we measured the rice-quality-related traits GT, GC, and AC in 173 different rice samples and conducted a genome-wide association study (GWAS) on these traits to identify the genes controlling eating quality. Our results provide insights into available genes and genetic resources for rice quality improvement, laying both a theoretical basis and a material framework for rice quality breeding.

## 2. Results

### 2.1. Phenotypic Variation in ECQ-Related Traits

**Figure 1:**
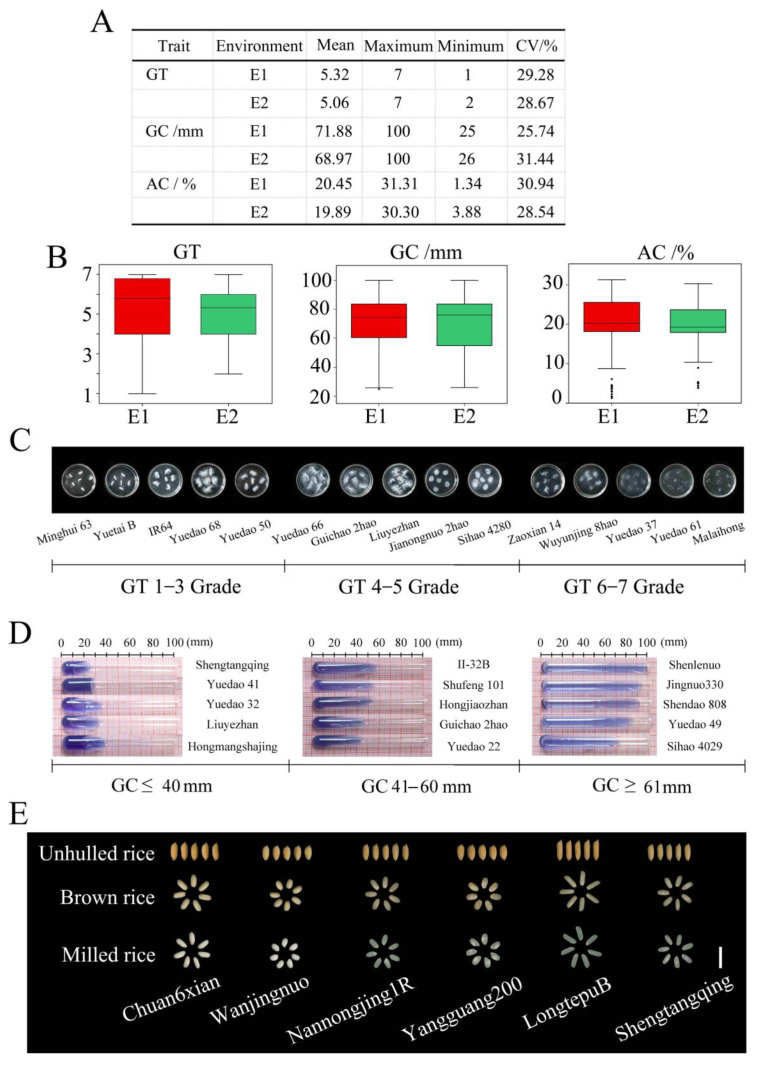
The phenotypic value description of ECQ-related traits among 173 rice accessions in two environments. (**A**) Basic statistics of ECQ-related traits in two environments. (**B**) Phenotypic value distributions of ECQ-related traits in two environments. The box edges indicate the upper and lower quantiles. The line in the middle of the box represents the median value. Vertical lines indicate the data from the lowest quantile to the top quantile. (**C**–**E**) The performance of GT (**C**), GC (**D**), and AC (**E**) in rice cultivars.

The basic statistics of the mean values, maximums, minimums, and coefficients of variation (CVs) of GT, GC, and AC are listed in Figure 1A. For the GT trait, the mean value was 5.19 in two environments, E1 and E2, with the CV ranging from 28.67% to 29.28% (Figure S1A). Figure 1B shows the GT distribution of the 173 samples in the E1 and E2 environments. The Minghui 63, Chuan 6xian, and Ningjinghui 260 varieties had high GTs, while Yuedao 37, Jindao 1007, and Zhongjing 131 had low GTs. The grade of GT was 6–7 (Figure 1C). For the GC trait, the mean values ranged from 68.97 ± 21.68 mm to 71.88 ± 18.50 mm. The CV was 28.59% in the two environments (Figure 1A). The GC distribution of the 173 samples in the E1 and E2 environments is shown in Figure 1B and Figure S1B. The samples of Liuyezhan, Malaihong, and Yuedao 41 had a hard GC of ≤40 mm (Figure 1D). The samples of Guichao 2hao, Nannongjing 1R, and Arias had a middle GC of 41–60 mm (Figure 1D). The samples of Shenlenuo, Zhongjing 131, and Luohanhuang had a soft GC of ≥61 mm (Figure 1D). For the AC trait, the maximum value was 31.31%, and the minimum value was 1.34% across the two environments (Figure 1A and Figure S1C). The CV ranged from 28.54% to 30.94% (Figure 1A). Figure 1B shows the AC distribution of 173 accessions in the two environments. The Shengtangqing variety had the highest AC of 29.34%. The Lincangwazuhangu variety had the lowest AC of 3.07%. The unhulled rice, brown rice, and milled rice varieties with different ACs are displayed in Figure 1E. These results indicate that there are abundant phenotypic variations in GT, GC, and AC among the 173 accessions, laying a foundation for mining elite variations related to GT, GC, and AC.

### 2.2. Identification of QTLs for ECQ-Related Traits Using GWAS

**Figure 2:**
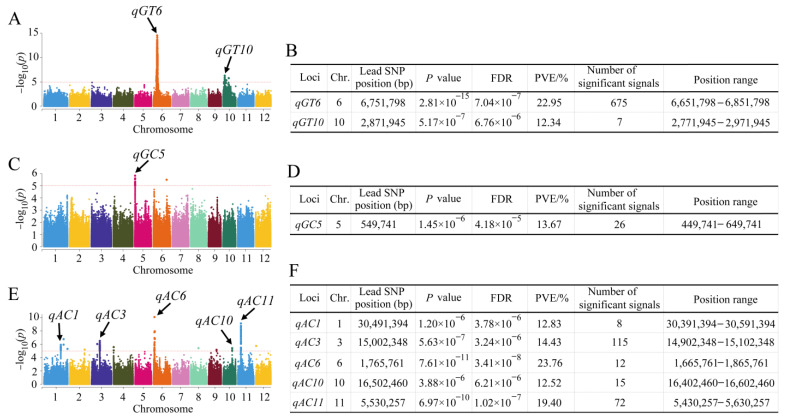
QTLs identified for GT, GC, and AC through GWAS in rice. (**A**) Manhattan plots for GT of the whole population of 173 rice accessions. (**B**) Information about the identified QTLs for GT. (**C**) Manhattan plots for GC of the whole population of 173 rice accessions. (**D**) Information about the identified QTLs for GC. (**E**) Manhattan plots for AC of the whole population of 173 rice accessions. (**F**) Information about the identified QTLs for AC. Negative log10 transformed *p*-values are plotted on the vertical axis, and dots above the red dashed line show the significant SNPs in the QTL region. The QTLs identified are shown by the black arrows. FDR, false discovery rate; PVE, phenotypic variation explained.

To investigate the possible natural variation in the ECQ of rice, a GWAS was conducted based on the association between the phenotype data of ECQ-related traits and 1,224,254 SNPs. We referred directly to the results of the population structure reported by Hu et al. [40]. Manhattan plots were constructed to show the significant SNPs related to rice ECQ. For the GT trait, two QTLs were detected and located on chromosomes 6 and 10 with the phenotypic variation explained (PVE) ranging from 12.34% to 22.95% (Figure 2A,B). There were 675 significant SNPs in the *qGT6* region. For the GC trait, only one QTL, *qGC5*, was identified and the PVE was 13.67% (Figure 2C,D). There were 26 significant SNPs in the *qGC5* region. For the AC trait, a total of five QTLs were identified, located on chromosomes 1, 3, 6, 10, and 11 (Figure 2E,F). The PVE ranged from 12.52% to 23.76%. There were 115 significant SNPs in the *qAC3* region. *qAC6* had the largest peak value (10.12), followed by *qAC11* (9.16).

Compared with the QTLs/genes reported previously, we found that the *ALK* gene, as reported by Gao et al. [41], overlapped within the *qGT6* QTL; the *Wx* gene, as reported by Zhang et al. [42] and Huang et al. [43], overlapped within the *qAC6 QTL.* The other six QTLs, *qGT10*, *qGC5*, *qAC1*, *qAC3*, *qAC10,* and *qAC11*, were newly found in this study. The functions of *ALK* and *Wx* have been well studied, so we will not analyze them in depth. Given that there were 72 significant SNPs in the *qAC11* region, and *qAC11* had a large peak value (9.16) and a PVE of 19.40%, we considered *qAC11* as a major QTL for AC. Next, we performed an analysis of *qAC11*.

### 2.3. Identification of Candidate Genes

**Figure 3:**
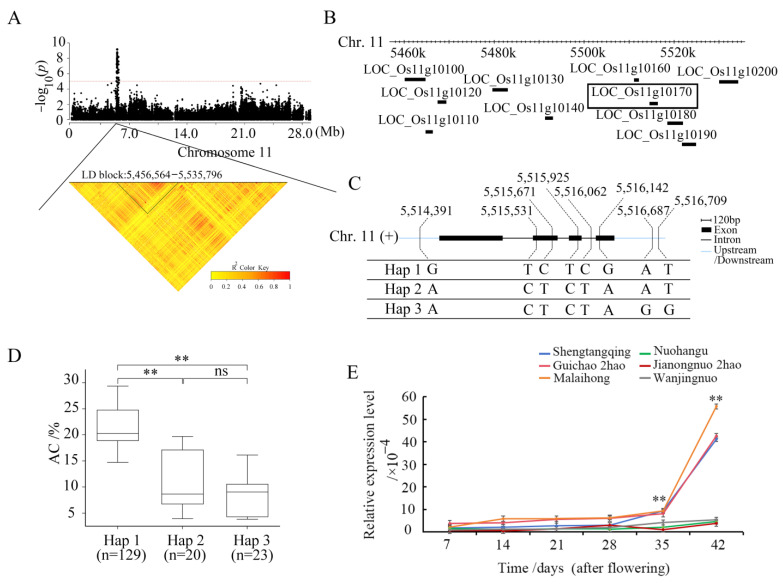
Identification of the candidate genes for the QTL *qAC11* for AC as determined by integrated analyses of GWAS and gene expression data in rice. (**A**) Identification of LD block of *qAC11*. Y axis means negative log10 transformed *p*-values and dots above the red line show the significant SNPs in the QTL region. Pairwise LD was determined by calculation of *r*^2^ (the square of the correlation coefficient between SNP states). (**B**) Identification of candidate genes in the region of *qAC11*. (**C**) Haplotypes of *LOC_Os11g10170* associated with AC in rice. (**D**) Box-plots of AC in accessions containing the different haplotypes. (**E**) Expression analysis of candidate gene *LOC_Os11g10170* in six materials with different AC values at different periods after flowering. The relative expression values were normalized to the rice UBQ gene. Error bars indicate standard deviation, and asterisks indicate significant differences using Student’s *t*-test (** *p* < 0.01). ns means no significance.

The potential candidate genes within 200 kb were analyzed in the chromosomal region containing *qAC11*. Twenty-eight positional candidate genes were identified in the *qAC11* region. Based on the LD analysis, the LD block region was determined to be 5,456,564–5,535,796 bp, containing nine candidate genes (Figure 3A,B). According to the SNP information, six of the nine genes contained nonsynonymous SNPs (Tables S1 and S2). Among the six genes, three genes (*LOC_Os11g10170*, *LOC_Os11g10180,* and *LOC_Os11g10200*) contained nonsynonymous SNPs significantly associated with AC (Tables S1 and S2).

The full length of *LOC_Os11g10170* was 1717 bp, which included four exons and three introns (Figure 3C). The *LOC_Os11g10170* gene encodes a flavin monooxygenase protein containing 387 amino acids. The SNPs occurred in the upstream, downstream, intron, and coding sequence of the gene, and three haplotypes were identified (Figure 3C). The haplotype Hap 1 was associated with a larger AC, and the average AC values of 129 accessions were 21.69 ± 3.84% (Figure 3D). The haplotypes Hap 2 and Hap 3 were associated with a smaller AC, and the average AC values for Hap 2 and Hap 3 were 8.60 ± 3.69% and 10.92 ± 5.56%, respectively (Figure 3D). There were highly significant differences between Hap 1 and Hap 2/Hap 3 at *p* < 0.01, and there were no significant differences between Hap 2 and Hap 3 (Figure 3D). The SNP site (5,515,531) caused a change from base T to base C at nt 649 within the cDNA sequence, resulting in an amino acid change from tryptophan (Trp) to arginine (Arg) at amino acid 217. The SNP site (5,516,142) caused a change from base G to base A at nt 1006 in the cDNA sequence, resulting in an amino acid change from aspartic acid (Asp) to asparagine (Asn) at amino acid 336.

The full length of *LOC_Os11g10180* was 3349 bp, including three exons and two introns. The *LOC_Os11g10180* gene encodes a 425-amino acid protein. The SNPs occurred in the 3′ UTR, the intron, and the coding sequence of the gene (Figure S2A). Three haplotypes were detected, but there were no significant differences between them (Figure S2B). The average AC value of 128 accessions for Hap 1 was 20.56 ± 5.77%. The average AC values for Hap 2 and Hap 3 were 18.80 ± 7.56% and 19.56 ± 1.46%, respectively (Figure S2B).

The base pairs of *LOC_Os11g10200* was 2068 bp, including five exons and four introns. The *LOC_Os11g10200* gene encodes a 436-amino acid protein. The SNPs occurred in the intron and coding sequence of the gene (Figure S3A). Two haplotypes were identified, but there were no significant differences between Hap 1 and Hap 2 (Figure S3B). The average AC values for Hap 1 and Hap 2 were 20.75 ± 4.85% and 18.12 ± 7.90%, respectively (Figure S3B).

The relative expression levels of three candidate genes in the endosperm between three accessions (Shengtangqing, Guichao 2hao, and Malaihong) with high ACs and three accessions (Nuohangu, Jianongnuo 2hao, and Wanjingnuo) with low ACs were analyzed on different days after flowering using qRT-PCR. The qRT-PCR results showed no significant differences in expression between the three candidate genes, except for the *LOC_Os11g10170* gene (Figure 3E, Figures S2C and S3C). The expression level of *LOC_Os11g10170* in high-AC accessions carrying Hap 1 was not significantly different from that in low-AC accessions carrying Hap 2/Hap 3 from 7d to 28d after flowering. The expression level of *LOC_Os11g10170* in high-AC accessions was significantly higher (*p* < 0.01) than that in low-AC accessions at 35d and 42d after flowering and reached the highest at 42d after flowering (Figure 3E). The expression level of the *GBSSI* gene was also checked. The results show that there were no significant differences in expression between the accessions with high ACs and those with low ACs at 7d, 28d, 35d, and 42d, except for 14d and 21d after flowering (Figure S4). At 14d after flowering, the difference in expression reached the maximum, which was similar to that reported by Zhang et al. [42]. These results suggest that enhanced expression of *LOC_Os11g10170* may increase AC.

## 3. Discussion

With the improvement of living standards, consumers show an increasing demand for the quality of rice, especially its eating quality. The ECQ is the most important trait in the evaluation of rice quality, determining the taste of rice. In breeding work, it is necessary to study the ECQ trait to select the hybrid offspring of rice more effectively. In this study, the phenotypic data of GT, GC, and AC in 173 accessions were investigated. The CV for GT, GC, and AC ranged from 25.74 to 31.44% (Figure 1). These results indicate a rich phenotypic variance, providing a material foundation for mining the elite variations for the ECQ trait.

Two QTLs for GT, one QTL for GC, and five QTLs for AC were identified (Figure 2). The *qGT6* QTL overlapped with the chromosomal location of the *ALK* gene reported by Gao et al. [41]. The *qAC6* QTL overlapped with the chromosomal location of the *Wx* gene reported by Zhang et al. [42]. The other six QTLs, *qGT10*, *qGC5*, *qAC1*, *qAC3*, *qAC10,* and *qAC11,* were newly found in this study, providing a molecular basis for genetically improving the ECQ trait.

Given the large peak value (9.16) and PVE (19.40%) of *qAC11*, we considered *qAC11* as a major QTL for AC. The potentially causal gene *AC11* was identified in the region of *qAC11*. Base substitutions within gene-coding regions, such as the change from base T to base C at nt 649, resulted in an amino acid change from Trp to Arg at amino acid 217. The changes of these amino acids may result in changes in the activity of amylose synthetase and affect the change in AC. The phenotypic value of accessions carrying the haplotype Hap 1 was 21.69 ± 3.84%, which was significantly higher than that of accessions carrying Hap 2 (8.60 ± 3.69%) and Hap 3 (10.92 ± 5.56%) (Figure 3D).

The *AC11* gene encodes YUCCA (YUC) flavin monooxygenases. There were 11 YUC genes in arabidopsis genomes and 14 YUC genes in rice genomes [44,45,46]. The gene effects of auxin on the regulation of endosperm development in arabidopsis and rice have been confirmed and reported [47,48,49,50,51]. Two YUC genes, *YUC10* (*At1g48910*) and YUC11 (*At1g21430*) in arabidopsis, and *OsYUC9* (*LOC_Os01g16714*), *OsYUC10* (*LOC_Os01g16750*), *OsYUC11* (*LOC_Os12g08780*), *OsYUC12* (*LOC_Os02g17230*), *OsYUC13* (*LOC_Os11g10140*), and *OsYUC14* (*LOC_Os11g10170*) in rice, were shown to play important roles in embryogenesis and endosperm development. The *AC11* gene was co-located with the *OsYUC14* gene. Meanwhile, *ZmYUC1* was confirmed as the key gene in the indole-3-acetic acid (IAA) biosynthesis in maize endosperm [52,53]. Overall, these results increase our understanding of the genetics of the IAA biosynthetic pathway.

Based on the results from both haplotype analysis and qRT-PCR, *LOC_Os11g10170* was considered as a candidate gene controlling AC. The expression level of *LOC_Os11g10170* in high-AC accessions was significantly higher (*p* < 0.01) than that in low-AC accessions at 35d and 42d after flowering and reached the highest at 42d after flowering (Figure 3E). These results provide a molecular basis for the further study of the function of *LOC_Os11g10170*.

In recent years, the complexity of starch synthesis and the variability in quality traits (susceptible to environmental influences) are important reasons for the lag in quality breeding. Besides genetic factors, environmental conditions are also one of the factors affecting rice eating quality [54,55]. A high temperature at the grain filling stage is an important factor in reducing rice eating quality. A high temperature not only causes the appearance of poor quality, but also leads to a decrease in the amylose content [56,57]. In general, conventional methods for improving rice quality are less efficient. The QTLs and genes detected in this study can provide theoretical guidance for rice quality improvement breeding, thereby improving the efficiency of quality breeding.

## 4. Materials and Methods

### 4.1. Plant Materials

A total of 173 *Oryza sativa* accessions were acquired for association mapping. The information on the 173 accessions, including names, origins, subpopulation classifications, and NCBI Sequence Read Archive accession numbers, are listed in Table S3. The seedlings of each accession were transplanted at the stage of four leaves with one heart in an experimental field at Hefei (N31.25°, E117.28°) for two years. Each material was planted in one plot. Each plot had 4 rows and 9 plants, with a spacing of 20 cm × 20 cm. Each accession was repeated twice in the field. Before testing, all the harvested grains of the 173 accessions were air-dried in a normal environment for at least three months.

### 4.2. Measurement of Rice GT, GC, and AC

After the rice seeds matured, six individual plants were randomly harvested in the middle of each plot. When the moisture content was approximately 14.5%, the individual plants were threshed. These unhulled rice grains were shelled and roughened with a machine (JLGJ4.5). The milled rice was obtained using a mini-type rice machine (JNMJ3, Taizhou, China). The rice flour was obtained with a high-speed universal crusher (FW100, Zhengzhou, China). After screening through a 100-mesh sieve, the rice flour was used to measure the quality traits.

The alkali digestion value (ADV) was used as the measurement index of GT via the alkali digestion test [58]. Six grains of milled rice were placed into a glass culture dish with a 6 cm diameter containing 10 mL of a 1.7% KOH solution. Then, the culture dish was laid into a 30 ± 0.5 °C incubator for approximately 23 h. After 23 h of incubation, the corresponding grade of each grain was recorded for each accession. Then, the mean value was calculated for the total grade of six grains. The mean value was used as the ADV of each accession. The ADVs were recorded as grades 1–7 [42]. Grade 1 showed the grain unchanged. Grade 2 showed the grain expansion. Grade 3 showed the grain expansion and ring intact and wide. Grade 4 showed the grain enlargement and ring intact and wide. Grade 5 showed the grain cracking and ring intact and wide. Grade 6 showed the grain partially dispersed, dissolved, and fused with the ring. Grade 7 showed the grain completely dispersed. Figure S5 displays the GT grades from 1 to 7 of GT. Grades 1–3 of GT belong to high GT. Grades 4–5 of GT belong to medium GT, and grades 6–7 of GT belong to low GT. The protocol of each variety was repeated twice, and the difference between the two replicates was less than 0.5. The mean value of two replicates was calculated as the GT of each variety.

According to the method of Cagampang et al. [59], the GCs of 173 varieties were measured. We collected 100 mg of rice flour into a glass tube and added 0.2 mL of a 0.025% thymol blue solution. After it fully dispersed, 2 mL of a 0.2 mol/L NaOH solution was added. Then, the tube was put into boiling water for heating for 8 min and then ice water for cooling for 20 min. After cooling, the tube was placed horizontally on a tray and put into an incubator to keep the temperature at 23 ± 2 °C for 1 h. After 1 h, the flow length of the rice gum was measured. A flow length of rice gum of less than 40 mm was considered as hard GC. A flow length of rice gum of 41–60 mm was considered as medium GC. A flow length of rice gum of more than 61 mm was considered soft GC. The protocol of each variety was repeated twice. For hard GC, the difference between the two replicates was less than 3 mm. For medium GC, the difference between the two replicates was less than 5 mm. For soft GC, the difference between the two replicates was less than 7 mm. The mean value of two replicates was calculated as the GC of each variety.

Based on the methods reported by Bao et al. [8], we collected 100 mg of rice flour into 100 mL capacity bottles. An amount of 1 mL of 95% ethanol was added to disperse the sample. After dispersion, 9 mL of a 1.0 mol/L NaOH solution was added. Then, the capacity bottles were put into boiling water for heating for 10 min and ice water for cooling for 30 min. After cooling to room temperature, distilled water was added to set the volume. An amount of 5 mL of the volumetric solution was transferred into another 100 mL capacity bottle. An amount of 1 mL of a 1 mol/L acetic acid solution and 1.5 mL of iodine solution were added to the bottle. Then, distilled water was added to set the volume, and the solution in the bottle was left for 20 min. An amount of 5 mL of the volumetric solution was transferred into another 100 mL capacity bottle. An amount of 5 mL of the 0.09 mol/L NaOH solution was taken to replace the sample, and the blank solution was prepared. The zero point of the photometer was adjusted at 620 nm with the blank solution. The absorbance of the colored sample was measured. Four standard samples of AC (1.5%, 10.4%, 16.2%, and 26.5%) were purchased from the China National Rice Research Institute and used to draw a standard curve. The regression equation for the standard curve was plotted as Y = a + b X, where Y is the amylose content of the sample, a is the intercept, b is the slope, and X is the absorbance value of the sample. Based on the absorbance value and regression equation, the AC of each variety was calculated. The protocol of each variety was repeated twice. The mean value of two replicates was calculated as the AC of each accession.

### 4.3. Genome-Wide Association Study

According to the description reported by Dang et al. [60], a total of 1,224,254 rice SNPs with a minor allele frequency of >5% and missing rates of <15% were selected for the genome-wide association study (GWAS). The software GAPIT v.2.12 was used to conduct the GWAS based on the compressed mixed linear model (MLM) program [61]. The Manhattan plot and Q–Q plot were generated with the qqman package of R [62]. The significance threshold of 1.0 × 10^−5^ for the GWAS was determined using the Benjamini and Hochberg [63] correction method. The software Haploview 4.2 was used to perform the linkage disequilibrium (LD) analysis and detect LD blocks surrounding significant SNPs [64]. The “LDheatmap” program of the R package was used to construct the LD heatmaps [65]. According to the description reported by Huang et al. [66], in these detected significant loci located on the same chromosome, we defined the leading SNP with the smallest *p*-value. If more than three SNPs exceeded the threshold line with a clear peak-like signal, these SNPs were divided into one QTL region. The length of the QTL region was 200 kb (100 kb upstream and 100 kb downstream of the leading SNP with the smallest *p*-value). Based on the LD analysis, the LD block was determined. Then the candidate gene was identified by combining the SNP information and gene annotation.

### 4.4. Candidate Gene Analysis

Based on the Nipponbare sequence from the MSU7.0 database, the candidate genes within a 200 kb genomic region were predicted. The SNP types for each candidate gene located in the candidate region were analyzed. Finally, the cause gene was determined based on the gene annotations and expression levels of the candidate genes.

### 4.5. Quantitative Reverse Transcription PCR Analysis of Candidate Genes

The grains after flowering for 7d, 14d, 21d, 28d, 35d, and 42d were sampled from three accessions with high AC values and three accessions with low AC values, respectively. The total RNA was extracted using the Magen Pure Plant Plus Kit (Magen Biotech Co., Ltd., Guangzhou, China). The first-strand cDNA was synthesized using the HisScript II Reverse Transcriptase system (Vazyme Biotech Co., Ltd., Nanjing, China). We used the UBQ rRNA gene as the internal control. The quantitative real-time polymerase chain reaction (qRT-PCR) was performed using SYBR Green (Vazyme Biotech Co., Ltd., Nanjing, China) based on the 96-well thermocycler (Thermo Fisher Scientific QuantStudio 6 Pro, Shanghai, China). The cycling conditions were set as follows: (1) denaturation (at 95 °C for 5 min); (2) an amplification and quantification program with 40 cycles (at 95 °C for 10 s, 60 °C for 30 s, and 72 °C for 60 s); (3) a melting curve (at 60–95 °C with a heating rate of 0.1 °C s^−1^); and (4) a cooling step (at 40 °C). Three biological replicate experiments were carried out for each sample, and the corresponding primers used for qRT-PCR are listed in Table S4. The transcript levels of gene expression were calculated following the method reported by Livak and Schmittgen [67]. The formula was as follows: the levels of gene expression = 2^−ΔΔCt^, where ΔCt = Ct_target gene_ − Ct_UBQ rRNA_.

### 4.6. Haplotype Analysis

We identified the haplotypes of candidate genes according to the databases of the China Rice Data Center (https://www.ricedata.cn/, accessed on 10 October 2023) and RiceVarMap (http://ricevarmap.ncpgr.cn/, accessed on 15 October 2023). Each haplotype was carried by at least 20 accessions.

### 4.7. Statistical Analysis

Microsoft Excel was used for statistical analysis (mean and standard error), graphing and Student’s *t* test.

## Data Availability

The datasets used and/or analyzed during the current study are available from the corresponding author upon reasonable request.

## Supplementary Materials

The following supporting information can be downloaded at: https://www.mdpi.com/article/10.3390/ijms25010630/s1.

## Abbreviations

AC: Amylose content; ADV: Alkali digestion value; ALK: Alkali digestion; Arg: Arginine; Asp: Aspartic acid; Asn: Asparagine; CV: Coefficient of variation; DBE: Debranching enzyme; ECQ: Eating-cooking quality; FDR: False discovery rate; Flo1: Floury endosperm 1; GBSS: Granule-bound starch synthase; GC: Gel consistency; GT: Gelatinization temperature; GWAS: Genome-wide association study; IAA: Indole-3-acetic acid; LD: Linkage disequilibrium; MLM: Mixed linear model; LSD1: Lesions stimulating disease resistance 1; OsEBP89: Oryza sativa Ethylene-responsive element binding protein 89; PVE: Phenotypic variation explained; qRT-PCR: Quantitative real-time polymerase chain reaction; QTL: Quantitative trait locus; RSR1: Rice starch regulator 1; SBE: Starch branching enzyme; SNP: Single nucleotide polymorphism; SSI: Starch synthase I; SSIIa: Starch synthase IIa; SSRG: Starch synthesis-related genes; Trp: Tryptophan; Wx: Waxy.

## References

[1] Juliano B.O., Brady N.C. (1979). The chemical basis of rice grain quality. Proceedings on the Workshop on Chemical Aspects of Rice Grain Quality.

[2] Xu C.W., Mo H.D. (1996). Qualitative-quantitative analysis for Inheritance of gelatinization temperature in Indica rice (*Oryza sativa* subsp. indica). Acta Agron. Sin..

[3] Umemoto T., Yano M., Satoh H., Shomura A., Nakamura Y. (2002). Mapping of a gene responsible for the difference in amylopectin structure between japonica-type and indica-type rice varieties. Theor. Appl. Genet..

[4] Gao Z.Y., Zeng D.L., Cheng F.M., Tian Z.X., Guo L.B., Su Y., Yan M.X., Jiang H., Dong G.J., Huang Y.C. (2011). ALK, the key gene for gelatinization temperature, is a modifier gene for gel consistency in rice. J. Integr. Plant Biol..

[5] Zhang G.Y., Cheng Z.J., Zhang X., Guo X.P., Su N., Jiang L., Mao L., Wan J.M. (2011). Double repression of soluble starch synthase genes SSIIa and SSIIIa in rice (*Oryza sativa* L.) uncovers interactive effects on the physicochemical properties of starch. Genome.

[6] Umemoto T., Aoki N. (2005). Single-nucleotide polymorphisms in rice starch synthase IIa that alter starch gelatinisation and starch association of the enzyme. Funct. Plant Biol..

[7] Fan M.Y., Wang X.J., Wang X.H., Tang L., Xu Q., Xu Z.J. (2017). Research advances of amylopectin structure in rice. Chin. J. Rice Sci..

[8] Bao J.S., Corke H., Sun M. (2006). Nucleotide diversity in Starch synthase IIa and validation of single nucleotide polymorphisms in relation to starch gelatinization temperature and other physicochemical properties in rice (*Oryza sativa* L.). Theor. Appl. Genet..

[9] Waters D., Henry R.J., Reinke R.F., Fitzgeraid M.A. (2006). Gelatinization temperature of rice explained by polymorphisms in starch synthase. Plant Biotechnol. J..

[10] Fujita N., Yoshida M., Asakura N., Ohdan T., Miyao A., Hirochika H., Nakamyra Y. (2006). Functional and characterization of starch synthase I using mutants in rice. Plant Physiol..

[11] Yang B.W., Xiang X.C., Xu S.J., Xu L., Wang Q. (2017). Effects for interaction of Wx and SSIII-2 on rice eating and cooking qualities. Acta Bot. Boreali-Occident Sin..

[12] Vandeputte G.E., Delcour J.A. (2004). From sucrose to starch granule to starch physical behaviour: A focus on rice starch. Carbohyd. Polym..

[13] Tian Z.X., Qian Q., Liu Q.Q., Yan M., Liu X., Yan C.J., Liu G., Gao Z., Tang S., Zeng D. (2009). Allele diversities in rice starch biosynthesis lead to a diverse array of rice eating and cooking qualities. Proc. Natl. Acad. Sci. USA.

[14] Zhu J.H., Zhang C.Q., Gu M.H., Liu Q.Q. (2015). Progress in the allelic variation of Wx gene and it’s application in rice breeding. Chin. J. Rice Sci..

[15] Zhang C.Q., Zhao D.S., Li Q.F., Gu M.H., Liu Q.Q. (2016). Progresses in research on cloning and functional analysis of key genes involving in rice grain quality. Sci. Agric. Sin..

[16] Zhang C.Q., Zhu J.H., Chen S.J., Fan X.L., Lu Y., Wang M., Yu H.X., Yi C.D., Tang S.Z., Gu M.H. (2019). Wx^lv^, the ancestral allele of rice Waxy gene. Mol. Plant.

[17] Wanchana S., Toojinda T., Tragoonrung S., Vanavichit A. (2003). Duplicated coding sequence in the waxy allele of tropical glutinous rice (*Oryza sativa* L.). Plant Sci..

[18] Wang Z.Y., Zheng F.Q., Shen G.Z., Gao J.P., Snustad D.P., Li M.G., Zhang J.L., Hong M.M. (1995). The amylose content in rice endosperm is related to the post-transcriptional regulation of the waxy gene. Plant J..

[19] Mikami I., Uwatoko N., Ikeda Y., Yamaguchi J., Hirano H.Y., Suzuki Y., Sano Y. (2008). Allele diversification at the wx locus in landraces of Asian rice. Theor. Appl. Genet..

[20] Chen Z.Z., Yang Y., Feng L.H., Sun Y., Zhang C.Q., Fan X.L., Li Q.F., Liu Q.Q. (2020). Effect of different combinations of Wx and ALK main alleles on rice grain quality. Chin. J. Rice Sci..

[21] Yan C.J., Tian S., Zhang Z.Q., Han Y.P., Chen F., Li X., Gu M.H. (2005). The source of genes related to rice grain starch synthesis among cultivated varieties and its contribution to quality. Sci. Agric. Sin..

[22] Zhu L.J., Gu M.H., Meng X.L., Cheung C.K., Yu H.X., Huang J., Sun Y., Shi Y.C., Liu Q.Q. (2012). High-amylose rice improves indices of animal health in normal and diabetic rats. Plant Biotechnol. J..

[23] Zhao H., Wang J.M., Zhang Q.F., Zhao Q., Mei S.F., Liu X.L., Cheng F.M. (2015). Activities of several starch synthesis enzymes in filling grains for rice sugary endosperm mutant (Sug-11) and it’s relation to starch quality. Chin. J. Rice Sci..

[24] Satoh H., Omura T. (1981). New endosperm mutations induced by chemical mutagens in rice *Oryza sativa* L.. Jpn. J. Breeding.

[25] She K.C., Kusano H., Koizumi K., Yamakawa H., Hakata M., Imamura T., Fukuda M., Naito N., Tsurumaki Y., Yaeshima M. (2010). A novel factor *FLOURY ENDOSPERM 2* is involved in regulation of rice grain size and starch quality. Plant Cell.

[26] Nishio T., Iida S. (1993). Mutants having a low content of 16-kDa allergenic protein in rice (*Oryza sativa* L.). Theor. Appl. Genet..

[27] Kang H.G., Park S., Matsuoka M., An G. (2005). White-core endosperm floury endosperm 4 in rice is generated by knockout mutations in the C4-type pyruvate orthophosphate dikinase gene (OsPPDKB). Plant J..

[28] Fujita N., Yoshida M., Kondo T., Saito K., Utsumi Y., Tokunage T., Nishi A., Satoh H., Park J., Jane J. (2007). Characterization of SSIIIa-deficient mutants of rice: The function of SSIIIa and pleiotropic effects by SSIIIa deficiency in the rice endosperm. Plant Physiol..

[29] Ryoo N., Yu C., Park C.S., Baik M.Y., Park I.M., Cho M.H., Bhoo S.H., An G., Hahn T., Jeon J.S. (2007). Knockout of a atarch synthase gene OsSSIIIa/Flo5 causes white-core floury endosperm in rice (*Oryza sativa* L.). Plant Cell Rep..

[30] Cheng P., Wang Y.H., Liu F., Ren Y.L., Zhou K.N., Lv J., Zheng M., Zhao S.L., Zhang L., Wang C.M. (2014). *FLOURY ENDOSPERM 6* encodes a CBM48 domain-containing protein involved in compound granule formation and starch synthesis in rice endosperm. Plant J..

[31] Fang P.F., Li S.F., Jiao G.A., Xie L.H., Hu P.S., Wei X.J., Tang S.Q. (2014). Physicochemical property analysis and gene mapping of a floury endosperm mutant flo7 in rice. Chin. J. Rice Sci..

[32] Sheng Z.H., Fang P.F., Li S.F., Jiao G.A., Xie L.H., Hu P.S., Tang S.Q., Wei X.J. (2015). Phenotype of rice floury endosperm mutant flo7 and fine mapping of mutated gene. Rice Sci..

[33] Silver D.M., Kötting O., Moorhead G.B.G. (2014). Phosphoglucan phosphatase function sheds light on starch degradation. Trends Plant Sci..

[34] Zhu Y., Cai X.L., Wang Z.Y., Hong M.M. (2003). An interaction between a MYC protein and an EREBP protein is involved in transciptional regulation of the rice Wx gene. J. Biol. Chem..

[35] Liu D.R., Huang W.X., Cai X.L. (2013). Oligomerization of rice granule-bound starch synthase 1 modulates its activity regulation. Plant Sci..

[36] Zhang H., Duan L., Dai J.S., Zhang C.Q., Li J., Gu M.H., Liu Q.Q., Zhu Y. (2013). Major QTLs reduce the deleterious effects of high temperature on rice amylose content by increasing splicing efficiency of Wx pre-mRNA. Theor. Appl. Genet..

[37] Fu F.F., Xue H.W. (2010). Coexpression analysis identifies rice starch regulator1, a rice AP2/EREBP family transcription factor, as a novel rice starch biosynthesis regulator. Plant Physiol..

[38] Wang J.C., Xu H., Zhu Y., Liu Q.Q., Cai X.L. (2013). OsbZIP58, a basic leucine zipper transcription factor, regulates starch biosynthesis in rice endosperm. J. Exp. Bot..

[39] Wu J.H., Zhu C.F., Pang J.H., Zhang X.R., Yang C.L., Xia G.X., Tian Y.C., He C.Z. (2014). OsLOL1, a C2C2-type zinc finger protein, interacts with OsbZIP58 to promote seed germination through the modulation of gibberellin biosynthesis in Oryza sativa. Plant J..

[40] Hu C.M., Jiang J.H., Li Y.L., Song S.J., Zou Y., Jing C.Y., Zhang Y., Wang D.Z., He Q., Dang X.J. (2022). QTL mapping and identification of candidate genes using a genome-wide association study for heat tolerance at anthesis in rice (*Oryza sativa* L.). Front. Genet..

[41] Gao Z.Y., Zeng D.L., Cui X., Zhou Y.H., Yan M.X., Huang D.N., Li J.Y., Qian Q. (2003). Map-based cloning of ALK gene, which controls the gelatinization temperature of rice. Sci. Chi. (Ser. C).

[42] Zhang C.Q., Yang Y., Chen S.J., Liu X.J., Zhu J.H., Zhou L.H., Lu Y., Li Q.F., Fan X.L., Tang S.Z. (2021). A rare Waxy allele coordinately improves rice eating and cooking quality and grain transparency. J. Integr. Plant Biol..

[43] Huang X.R., Su F., Huang S., Mei F.T., Niu X.M., Ma C.L., Zhang H., Zhu X.G., Zhu J.K., Zhang J.S. (2021). Novel Wx alleles generated by base editing for improvement of rice grain quality. J. Integr. Plant Biol..

[44] Yamamoto Y., Kamiya N., Morinaka Y., Matsuoka M., Sazuka T. (2007). Auxin biosynthesis by the YUCCA genes in rice. Plant Physiol..

[45] Zhang T., Li R.N., Xing J.L., Yan L., Wang R.C., Zhao Y.D. (2018). The YUCCA-Auxin-WOX11 module controls crown root development in rice. Front. Plant Sci..

[46] Xu X.Y., E Z.G., Zhang D.P., Yun Q.B., Zhou Y., Niu B.X., Chen C. (2021). OsYUC11-mediated auxin biosynthesis is essential for endosperm development of rice. Plant Physiol..

[47] Cheng Y.F., Dai X.H., Zhao Y.D. (2006). Auxin biosynthesis by the YUCCA flavin monooxygenases controls the formation of floral organs and vascular tissues in Arabidopsis. Genes Dev..

[48] Cheng Y.F., Dai X.H., Zhao Y.D. (2007). Auxin synthesized by the YUCCA flavin monooxygenases is essential for embryogenesis and leaf formation in *Arabidopsis*. Plant Cell.

[49] Abu-Zaitoon Y.M., Bennett K., Normanly J., Nonhebel H.M. (2012). A large increase in IAA during development of rice grains correlates with the expression of trytophan aminotransferase OsTAR1 and a grain specific YUCCA. Physiol. Plant.

[50] Chen Q.G., Dai X.H., De-Paoli H., Cheng Y., Takebayashi Y., Kasahara H., Kamiya Y., Zhao Y.D. (2014). Auxin overproduction in shoots cannot rescue auxin deficiencies in Arabidopsis roots. Plant Cell Physiol..

[51] Zhang X.F., Tong J.H., Bai A.N., Liu C.M., Xiao L.T., Xue H.W. (2020). Phytohormone dynamics in developing endosperm influence rice grain shape and quality. J. Integr. Plant Biol..

[52] Bernardi J., Lanubile A., Li Q.B., Kumar D., Kladnik A., Cook S.D., Ross J.J., Marocco A., Chourey P.S. (2012). Impaired auxin biosynthesis in the defective endosperm 18 mutant is due to mutational loss of expression in the ZmYuc 1 gene encoding endosperm-specific YUCCA1 protein in maize. Plant Physiol..

[53] Bernardi J., Battaglia R., Bagnaresi P., Lucini L., Marocco A. (2019). Transcriptomic and metabolomic analysis of ZmYUC1 mutant reveals the role of auxin during early endosperm formation in maize. Plant Sci..

[54] Champagne E.T., Bett-Garber K.L., Fitzgerald M.A., Grimm C.C., Lea J., Ohtsubo K.I., Jongdee S., Xie L.H., Bassinello P.Z., Resurreccion A. (2010). Important sensory properties differentiating premium rice varieties. Rice.

[55] Xu Y.J., Ying Y.N., Ouyang S.H., Duan X.L., Sun H., Jiang S.K., Sun S.C., Bao J.S. (2018). Factors affecting sensory quality of cooked japonica rice. Rice Sci..

[56] Sreenivasulu N., Butardo V.M.J., Misra G., Cuevas R.P., Anacleto R., Kavi Kishor P.B. (2015). Desinging climate-resilient rice with ideal grain quality suited for high-temperature stress. J. Exp. Bot..

[57] Zhang C.Q., Chen S.J., Ren X.Y., Lu Y., Liu D.R., Cai X.L., Li Q.F., Gao J.P., Liu Q.Q. (2017). Molecular structure and physicochemical properties of starches from rice with different amylose contents resulting from modification of OsGBSSI activity. J. Agric. Food Chem..

[58] Little R.R., Hiller G.B., Son E. (1958). Differential effect of dilute alkali on 25 varieties of milled white rice. Cereal Chem..

[59] Cagampang G.B., Perez C.M., Bo J.O. (1973). A gel consistency test for eating quality of rice. J. Sci. Food Agric..

[60] Dang X.J., Yang Y., Zhang Y.Q., Chen X.G., Fan Z.L., Liu Q.M., Ji J., Li D.L., Li Y.H., Fang B.J. (2020). *OsSYL2*^AA^, an allele identified by gene-based association, increases style length in rice (*Oryza sativa* L.). Plant J..

[61] Lipka A.E., Tian F., Wang Q.S., Peiffer J., Li M., Bradbury P.J., Gore M.A., Buckler E.S., Zhang Z.W. (2012). GAPIT: Genome association and prediction integrated tool. Bioinformatics.

[62] Turner S.D. (2014). qqman: An R package for visualizing GWAS results using QQ and Manhattan plots. BioRxiv.

[63] Benjamini Y., Hochberg Y. (1995). Controlling the false discovery rate: A practical and powerful approach to multiple testing. J. R. Stat. Soc. B.

[64] Barrett J.C., Fry B., Maller J., Daly M.J. (2005). Haploview: Analysis and visualization of LD and haplotype maps. Bioinformatics.

[65] Shin J.H., Blay S., McNeney B., Graham J. (2006). LDheatmap: An R function for graphical display of pairwise linkage disequilibria between single nucleotide polymorphisms. J. Stat. Softw..

[66] Huang Z.B., Ying J.F., Peng L.L., Sun S., Huang C.W., Li C., Wang Z.F., He Y.Q. (2021). A genome-wide association study reveals that the cytochrome b5 involved in seed reserve mobilization during seed germination in rice. Theor. Appl. Genet..

[67] Livak K.J., Schmittgen T.D. (2001). Analysis of relative gene expression data using real-time quantitative PCR and the 2^−ΔΔCt^ method. Methods.

