# Supplementary material for: QTL Detection and Candidate Gene Identification for Eating and Cooking Quality Traits in Rice (Oryza sativa L.) via a Genome-Wide Association Study
Source: Int J Mol Sci. 2024 Jan 3;25(1):630. doi: 10.3390/ijms25010630 (PMC10779416; doi:10.3390/ijms25010630)
Supplement: Supplementary file 1 [file ijms-25-00630-s001.zip › Figure S1-S5.pdf]

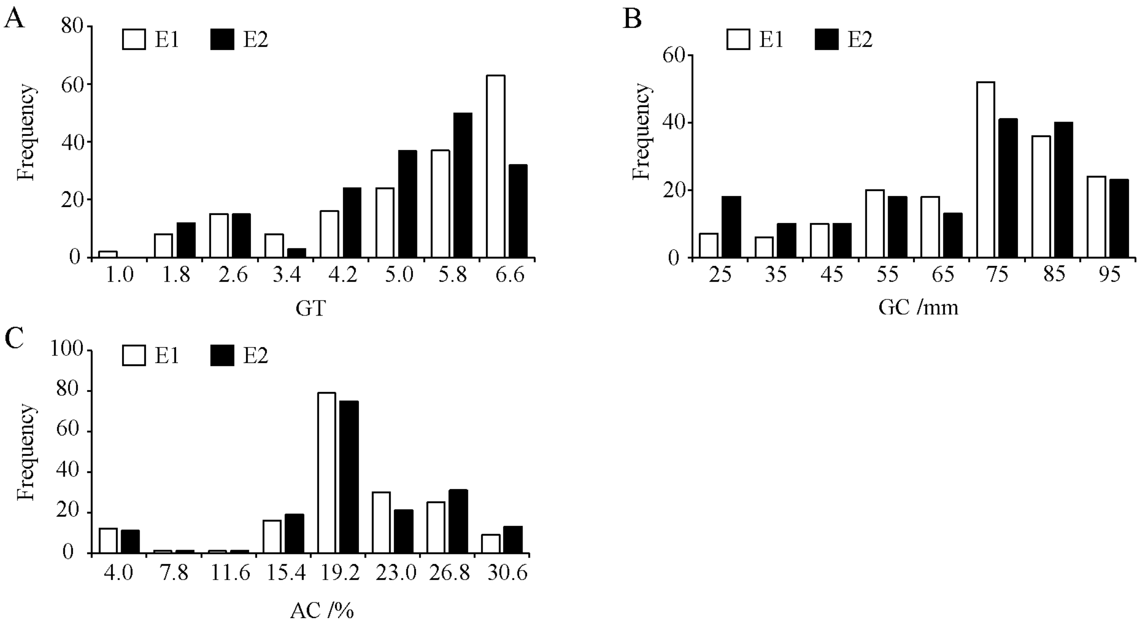

Figure S1. Diagram of all three traits in two environments. (A) GT in E1 and E2. (B) GC in E1 and E2. (C) AC in the E1 and E2. E1, environment 1; E2, environment 2; GT, gelatinization temperature; GC, gel consistency; AC, amylose content.

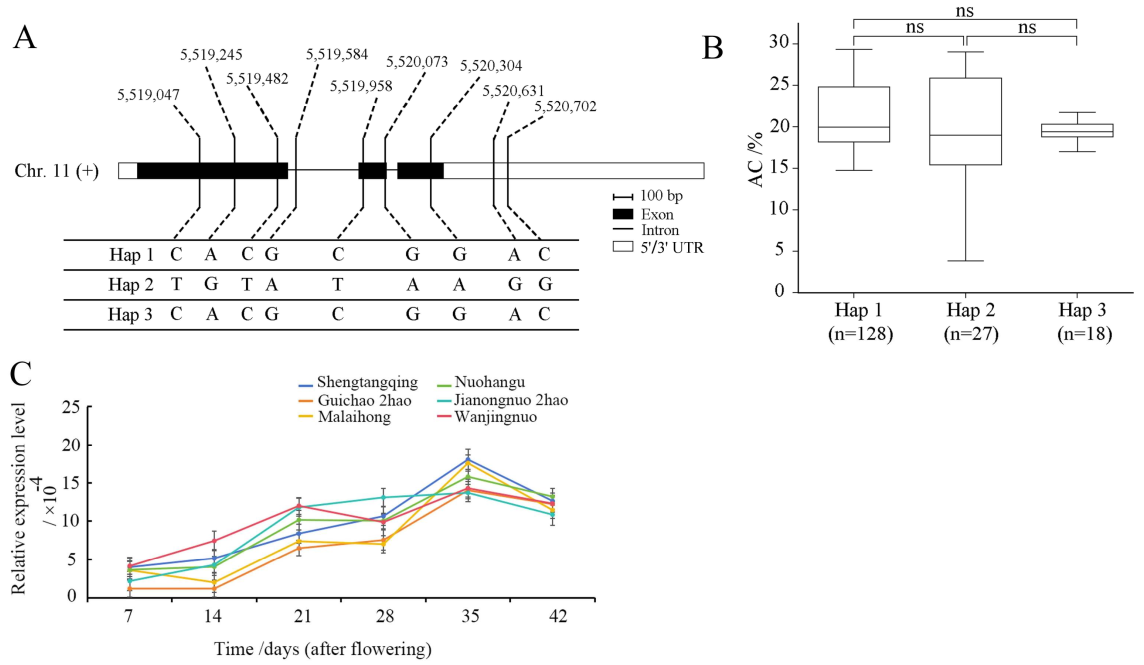

Figure S2. Identification of the candidate gene *LOC_Os11g10180* for AC. (A) Gene structure and haplotypes of LOC_Os11g10180. (B) Box-plots of AC in accessions containing the different haplotypes. (C) Expression analysis of candidate gene LOC_Os11g10180 in six materials with different AC value at different periods after flowering. The relative expression values were normalized to the rice UBQ gene. Error bars indicate standard deviation, and ns means no significance.

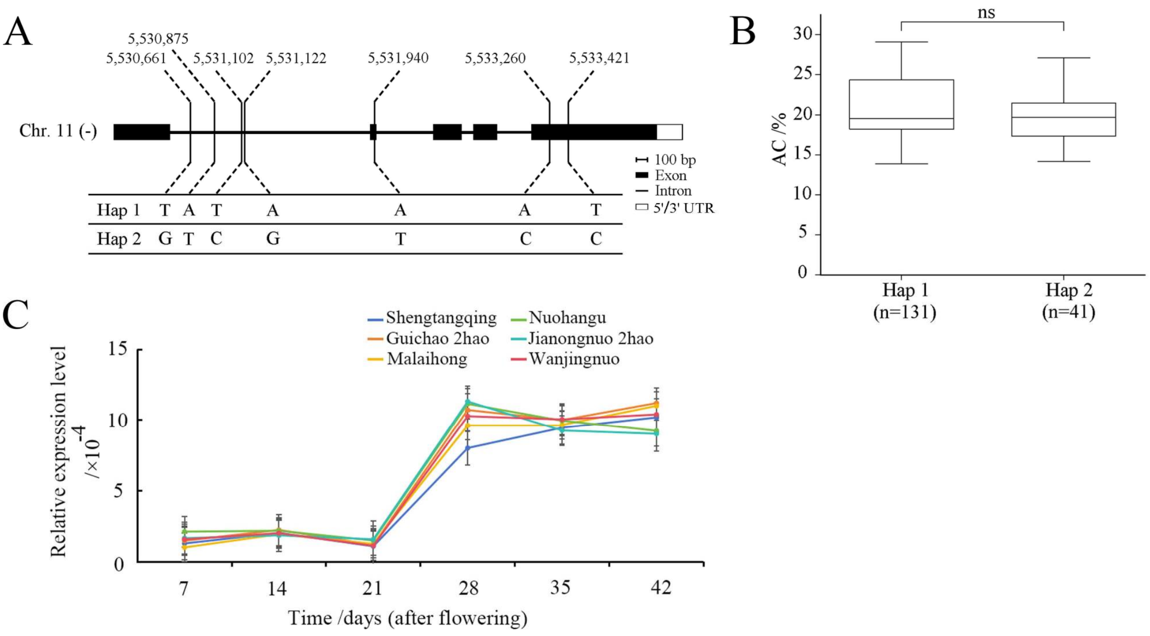

Figure S3. Identification of the candidate gene *LOC_Os11g10200* for AC. (A) Gene structure and haplotypes of LOC_Os11g10200. (B) Box-plots of AC in accessions containing the different haplotypes. (C) Expression analysis of candidate gene LOC_Os11g10200 in six materials with different AC value at different periods after flowering. The relative expression values were normalized to the rice UBQ gene. Error bars indicate standard deviation, and ns means no significance.

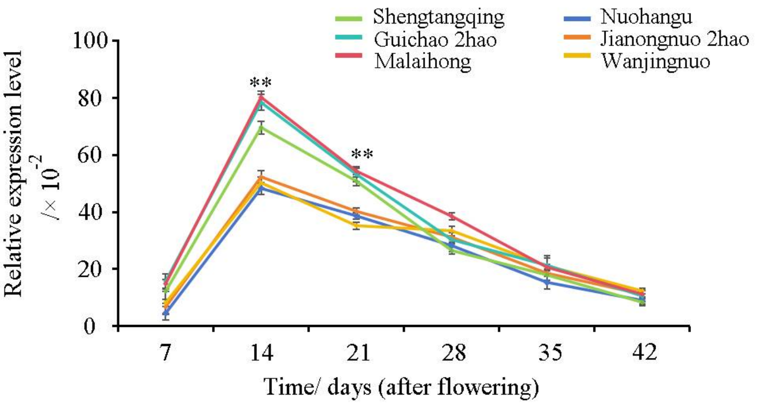

Figure S4. Expression analysis of gene *GBSSI* in six materials with different AC value at different periods after flowering. The relative expression values were normalized to the rice UBQ gene. Error bars indicate standard deviation, and asterisks indicate significant differences using the Student' s t-test (**P < 0.01).

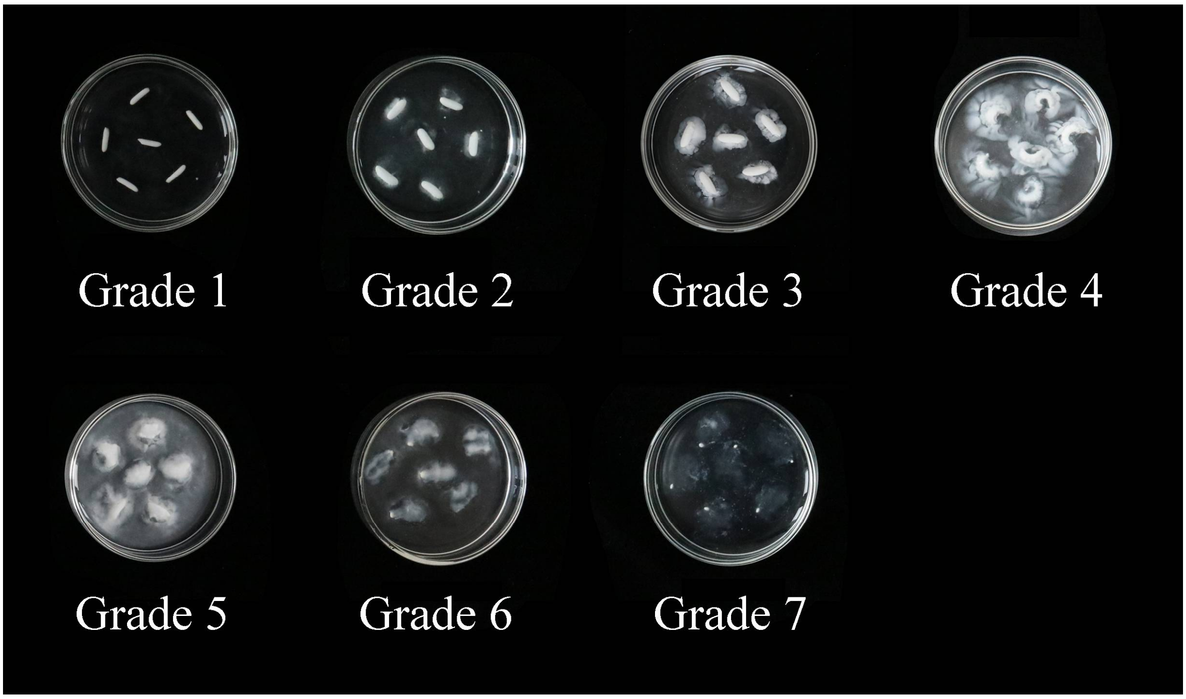

Figure S5. Grades from 1 to 7 of gelatinization temperature.
