# Supplementary material for: QTL Detection and Candidate Gene Identification for Eating and Cooking Quality Traits in Rice (Oryza sativa L.) via a Genome-Wide Association Study
Source: Int J Mol Sci. 2024 Jan 3;25(1):630. doi: 10.3390/ijms25010630 (PMC10779416; doi:10.3390/ijms25010630)
Supplement: Supplementary file 1 [file ijms-25-00630-s001.zip › Table S2.pdf]

Table S2. Candidate gene annotation in the region 5.46–5.54 Mb associated with amylose content.

Number	MSU ID	Position	Annotation
1	LOC_Os11g10100	5,460,201 - 5,464,655	Mitogen-activated protein kinase
2	LOC_Os11g10110	5,464,851 - 5,466,306	Expressed protein
3	LOC_Os11g10120	5,467,512 - 5,469,283	Expressed protein
4	LOC_Os11g10130	5,479,654 - 5,482,999	MYB family transcription factor
5	LOC_Os11g10140	5,491,381-5,493,004	Flavin monooxygenase
6	LOC_Os11g10160	5,511,173 - 5,512,067	Expressed protein
7	LOC_Os11g10170	5,514,584 - 5,516,300	Flavin monooxygenase
8	LOC_Os11g10180	5,518,581 -5,521,929	OsFBX412-F-box domain containing protein
9	LOC_Os11g10200	5,530,074-5,534,187	OsFBX413-F-box domain containing protein
