# Supplementary material for: QTL Detection and Candidate Gene Identification for Eating and Cooking Quality Traits in Rice (Oryza sativa L.) via a Genome-Wide Association Study
Source: Int J Mol Sci. 2024 Jan 3;25(1):630. doi: 10.3390/ijms25010630 (PMC10779416; doi:10.3390/ijms25010630)
Supplement: Supplementary file 1 [file ijms-25-00630-s001.zip › Table S4.pdf]

Table S4. The sequences of primers used for qRT-PCR.

Gene name	Forward primer sequence (5’-3’)	Reverse primer sequence (5’-3’)
UBQ	ACCCTGGCTGACTACAACATC	AGTTGACAGCCCTAGGGTG
LOC_Os11g10170	AGGGCTAAATGTGACAGCAG	GTGCCAACATCAATAACGGC
LOC_Os11g10180	GTTGGTGGCCTGGAAATGTG	TTCGAGAAACACGCAGTCGT
LOC_Os11g10200	TTCTCGGCATTGATACGGCA	GATTTCCCGGAGGACAAGGG
GBSSI/Wx	ATTCCTTCAGTTCTTTGTCTATCTCA	ATGGTGGTTGTCTAGCTGTTGC
